# Supplementary material for: Systematic Comparison of Plant Promoters in Nicotiana spp. Expression Systems
Source: Int J Mol Sci. 2022 Dec 6;23(23):15441. doi: 10.3390/ijms232315441 (PMC9740895; doi:10.3390/ijms232315441)
Supplement: Supplementary file 1 [file ijms-23-15441-s001.zip › Supplementary tables S1-S3.pdf]

Systematic comparison of plant promoters in *Nicotiana* spp. expression systems

Ekaterina S. Shakhova, Nadezhda M. Markina, Tatiana Mitiouchkina, Evgenia N. Bugaeva, Tatiana A. Karataeva, Kseniia A. Palkina, Liliia I. Fakhranurova, Ilia V. Yampolsky, Karen S. Sarkisyan, Alexander S. Mishin[*]

Supplementary information

Table S1. Level 1 vectors used in this study. **2**

Table S2. Level 0 plasmids with promoters and terminators used in this study. **4**

Table S3. Primers used in this study. **8**

Table S1. Level 1 vectors used in this study.

Type	ID	Structure	Sequence
Virus promoter	pNK955	Level1| **p35s_0.4kb** - 5'UTR TMV omega - EGFP - tOCS	https://benchling.com/s/seq-Xv4yj8acyzr5Ms9vWE92?m=slm-Z04z3oSgMyZVnFCU5oP3
	pNK1481	Level1 | **p35s_0.4kb** - 5'UTR AtRBCS2B - EGFP - tOCS	https://benchling.com/s/seq-NWaRFM9UiqVg3Kk62CD3?m=slm-yd34JT03rKFJl2FQ2FrR
	pX088	Level1 | **pFMV** - EGFP - tOCS	https://benchling.com/s/seq-IBV1TXfJMIvmcuLdRbTg?m=slm-PJ0oohwynpUw1tVppfWr
	pX089	Level1 | **pCmYLCV** - EGFP - tOCS	https://benchling.com/s/seq-BzVQwacCASJ3QiXyT5LS?m=slm-mGNqUdrifxQz9BzGYI1n
Synthetic promoter	pNK951	Level1 | **pMinSyn104** - 5'UTR AtRBCS2B - EGFP - tOCS	https://benchling.com/s/seq-0kYMTsYtcpEFoA5rVmsy?m=slm-3b27GbcFKiY6r614Pwxz
	pNK1513	Level1 | **pMinSyn105** - 5'UTR AtRBCS2B - EGFP - tOCS	https://benchling.com/s/seq-jgWZjw7OI5PjWVqSbXAh?m=slm-dR4dUfIhyHbZQS2LoZ4f
	pNK1141	Level1 | **pMinSyn108** - 5'UTR AtRBCS2B - EGFP - tOCS	https://benchling.com/s/seq-OS9XK2vW90gpwnk5SYAb?m=slm-vewEeMhX16F6R1HambEF
	pNK1137	Level1 | **pMinSyn110** - 5'UTR AtRBCS2B - EGFP - tOCS	https://benchling.com/s/seq-VpQIZc8dgtwbPd3iUmvX?m=slm-OLWrMFaWlEgMlhGlbtLx
	pNK952	Level1 | **pMinSyn159x** - 5'UTR AtRBCS2B - EGFP - tOCS	https://benchling.com/s/seq-s5B6tzYHfvqwUXx2UoVu?m=slm-qic0dr0QGeRLKlVGsKAi
	pNK1112	Level1 | **pMinSyn159x** - 5'UTR AtRBCS2B - EGFP - **tAct2**	https://benchling.com/s/seq-oYCEmyaF71RfBpTAbEFg?m=slm-riK7MJUwcGiEKw96thBA
	pNK1412	Level1 | **pMinSyn159x** - 5'UTR AtRBCS2B - EGFP - **tRBCS3C**	https://benchling.com/s/seq-ztYZj76YosOM2A2iNW1U?m=slm-DtHx6UiaPicCpug1FOyH
	pNK1236	Level1 | **pMinSyn159x** - 5'UTR AtRBCS2B - EGFP - **tATPase**	https://benchling.com/s/seq-aPufVoUSKizTaFhT2E3E?m=slm-NrKKtuxswEIZN1Ax1N82
	pNK1111	Level1 | **pMinSyn159x** - 5'UTR AtRBCS2B - EGFP - **tHSP18.2**	https://benchling.com/s/seq-wjSuFYeCnattBzyfJgW4?m=slm-Rl5kiSudgkfekJeObr49
	pNK1140	Level1 | **pMinSyn1556x** - 5'UTR AtRBCS2B - EGFP - tOCS	https://benchling.com/s/seq-hLOY4zQtQts9ICyJgKBs?m=slm-CGqSZUiNYxwabad0OfWO
	pNK1138	Level1-1 | **pMinSyn1569x** - 5'UTR AtRBCS2B - EGFP - tOCS	https://benchling.com/s/seq-19omt2Y0LD6NW9aeAK1G?m=slm-uflaYDAe812s51fEk8YE
	pNK1515	Level1 | **pMinSyn1637x** - 5'UTR AtRBCS2B - EGFP - tOCS	https://benchling.com/s/seq-SyrFSUGSJwj2uGYBEQ7A?m=slm-0kpvhriPePECa2OBKKm5
	pNK1036	Level1 | **pMinSyn1824x** - 5'UTR AtRBCS2B - EGFP - tOCS	https://benchling.com/s/seq-gyWD4RBezbwQxFdQl6rm?m=slm-t9BfguUZgXrirE5XYSQs
	pNK1037	Level1 | **pMinSyn1904x** - 5'UTR AtRBCS2B - EGFP - tOCS	https://benchling.com/s/seq-TU9mjtz0iPxozRGdvp0G?m=slm-tPn3qpoBsNjjcnyz6rUG

Plant promoter	pNK953	Level1 | **pAtTCTP** - EGFP - tOCS	https://benchling.com/s/seq-8fQeI0byzUI350uKtDYr?m=slm-EVEL4TI37GQodCF8wJq6
	pNK1145	Level1 | **pAtTCTP** - EGFP - **tAct2**	https://benchling.com/s/seq-rV4DWhZy2F8GIzrsT9NS?m=slm-BfB3mjNUvIIZ4Od4GDIb
	pNK1152	Level1 | **pAtTCTP** - EGFP - **tRBCS3C**	https://benchling.com/s/seq-8pJOK3HtycSzLCRpLpAz?m=slm-EjZVbezlO9ucYHnsBqNz
	pNK1143	Level1-1 | **pAtTCTP** - EGFP - **tATPase**	https://benchling.com/s/seq-YYeDtyIG69ZQrAqnUlyD?m=slm-waMZwW0kseP60KbNrp80
	pNK1114	Level1 | **pAtTCTP** - EGFP - **tHSP18.2**	https://benchling.com/s/seq-9kcntvkxWSNYzQyvoUuM?m=slm-Hrv8rcIAXuKELmlSuTbE
	pNK954	Level1 | **pdel5_MtHP** - EGFP - tOCS	https://benchling.com/s/seq-QvJzk8QaFH1e1O8jAYbI?m=slm-fXzAyVAzwBKLxr87DQNf
	pNK1110	Level1 | **pdel5_MtHP** - EGFP - **tAct2**	https://benchling.com/s/seq-ivePFin9Q5nAZf4C0VnD?m=slm-I44K4zbj9OhxGAS1PMTv
	pNK1109	Level1 | **pdel5_MtHP** - EGFP - **tRBCS3C**	https://benchling.com/s/seq-Y0p5GIBsbAcEV4hn84MI?m=slm-EEdXCcENRhGd7bJjCeQT
	pNK1113	Level1 | **pdel5_MtHP** - EGFP - **tATPase**	https://benchling.com/s/seq-l1VojRlnf0GEhwlh1f3m?m=slm-gVPTKyqzFagJyE41nb5z
	pNK1144	Level1 | **pdel5_MtHP** - EGFP - **tHSP18.2**	https://benchling.com/s/seq-StzkvqM01NeKpD4eTwmn?m=slm-yFiEIb0eKdoydeRkdEDL
	pNK931	Level1 | **pAtUBQ10** - EGFP - tOCS	https://benchling.com/s/seq-TzWi1PDZB5OVr2ws61GE?m=slm-mqXjDgtkE7aeSCsH399o
	pNK1106	Level1 | **pAtUBQ10** - EGFP - **tAct2**	https://benchling.com/s/seq-ApnWp2eG3BQdHu664IMh?m=slm-weypOv2dQBHSmaZvEk8l
	pNK1108	Level1 | **pAtUBQ10** - EGFP - **tRBCS3C**	https://benchling.com/s/seq-hVLNKYvVIK1qf11tVblC?m=slm-DDzyAFgWHYyiC2Fmyh5z
	pNK1107	Level1 | **pAtUBQ10** - EGFP - **tATPase**	https://benchling.com/s/seq-cvAPHCypUt5fYh2j2Wdu?m=slm-MTzgxDRKhI2tGC1YmooF
	pNK1142	Level1 | **pAtUBQ10** - EGFP - **tHSP18.2**	https://benchling.com/s/seq-twwPDIyKDsduzx0R0Dvc?m=slm-UykCIrMtTr0IlBWOL9O4
	pNK930	Level1 | **pAtRPS5a** - EGFP - tOCS	https://benchling.com/s/seq-ykQtGbOXM8pmSIZjgnNa?m=slm-C7lg4SOTyizdhjTqbfYu
	pNK932	Level1 | **pAtPD7** - EGFP - tOCS	https://benchling.com/s/seq-PXTVGBjI2QJHHahIFlDR?m=slm-wAJ0Fs0OlaLRHQeL5hUj
	pX083	Level1 | **pAtAct2** - 5'UTR TMV omega - EGFP - tOCS	https://benchling.com/s/seq-0ZfjzZWajg0osBliICcA?m=slm-5BlLr6n3iYcUyQEzrefo

Table S2. Level 0 plasmids with promoters and terminators used in this study.

ID	Name of the construct	Sequence	DNA part type	Source of plasmid
pICH51277<br>MoClo Plant Parts Kit<br>Addgene (Kit #1000000047)	Level 0 - promoter | **p35s_0.4kb** - 5'UTR TMV omega	https://www.addgene.org/50268/	PROM + 5UTR	[7]
pICH41388<br>MoClo Plant Parts Kit<br>Addgene (Kit #1000000047)	Level 0 - promoter | **p35s_0.4kb**	https://www.addgene.org/50253/	PROM	[7]
pNK883	Level 0 - promoter | **pMinSyn104**	https://benchling.com/s/seq-bwu4KtFbPI7C3f7wrm5m?m=slm-LX8478zw5MY3eK6LxsCS	PROM	[5]
pNK880	Level 0 - promoter | **pMinSyn105**	https://benchling.com/s/seq-mtPe4uNuJkUREyk1E9TB?m=slm-4P6pmRnEleWlYE24locP	PROM	
pNK879	Level 0 - promoter | **pMinSyn108**	https://benchling.com/s/seq-qahowDDKd9M3JvNHfeDg?m=slm-CGsjRUx8x3bmIA2Y4Oxn	PROM	
pNK887	Level 0 - promoter | **pMinSyn110**	https://benchling.com/s/seq-Cn85nhOm0I2DMeEDzHBO?m=slm-cp1QzkzwES1QmA7dk7c6	PROM	
pNK882	Level 0 - promoter | **pMinSyn159x**	https://benchling.com/s/seq-lAviApCq4kkds4HrfgNf?m=slm-ASMYEg0pM5bqFNVDpJqI	PROM	

pNK881	Level 0 - promoter | **pMinSyn1556x**	https://benchling.com/s/seq-lvkyH4I3dhhdUx569J8A?m=slm-bztFB6F96JxkqnhuDyP6	PROM	
pNK884	Level 0 - promoter | **pMinSyn1569x**	https://benchling.com/s/seq-fmxiLzQJdsRj4vd3332T?m=slm-mSlQsnDH5xkF3BDAwtKA	PROM	
pNK6030	Level 0 - promoter | **pMinSyn1637x**	https://benchling.com/s/seq-UhSCuiyG3wbeYAofc97i?m=slm-M1s4yJgnDkcb08MPlMTj	PROM	
pNK885	Level 0 - promoter | **pMinSyn1824x**	https://benchling.com/s/seq-JeaHhnuxRXrv2CifdrMi?m=slm-gNO7kcdpJJfknvoNcgvH	PROM	
pNK878	Level 0 - promoter | **pMinSyn1904x**	https://benchling.com/s/seq-Iw4GyN7iasauX3DyXzuS?m=slm-WcW504iKeOw6zwznAFHg	PROM	
pNK875	Level 0 - promoter + 5U | **pAtTCTP**	https://benchling.com/s/seq-cIKcYEcM2UbC2DJcZuiz?m=slm-dV56BTwMS6j3n0TySGfb	PROM + 5UTR	[14]
pNK928	Level 0 - promoter + 5U | **pdel5_MtHP**	https://benchling.com/s/seq-fMZgG4tPifg3OGQTTPVX?m=slm-Y5CtJNoPrUwOWApIRSTM	PROM + 5UTR	[15]
pJOG684<br>MoClo Plant Parts II and Infrastructure Kit<br>Addgene<br>(Kit #1000000135)	Level 0 - promoter + 5U | **pAtUBQ10**	https://www.addgene.org/105355/	PROM + 5UTR	[16]

pJOG603<br>MoClo Plant Parts II and Infrastructure Kit<br>Addgene<br>(Kit #1000000135)	Level 0 - promoter + 5U | **pAtRPS5a**	https://www.addgene.org/105356/	PROM + 5UTR	[16]
pNK869	Level 0 - promoter + 5U | **pAtPD7**	https://benchling.com/s/seq-CNIr9MZN9n6irNXQMYKb?m=slm-Rs0KA3oOcLiXcUoId34X	PROM + 5UTR	[17]
pICH87644<br>MoClo Plants Part Kit<br>Addgene (Kit #1000000047)	Level 0 - promoter + 5U | **pAtAct2 + 5'UTR TMV omega**	https://www.addgene.org/50274/	PROM + 5UTR	[7]
pNK6032	Level 0 - promoter + 5U | **pFMV**	https://benchling.com/s/seq-iXRBZ2D4pWOtMJyhjBJT?m=slm-B4U18SGhRBGriHoJlABC	PROM + 5UTR	[12]
pNK6031	Level 0 - promoter + 5U | **pCmYLCV**	https://benchling.com/s/seq-4oY2mNHqVNuj4XYIAkUY?m=slm-QUgDDSQAFmhUsuZLBvgI	PROM + 5UTR	[13]
pICH44179<br>MoClo Plants Part Kit<br>Addgene (Kit #1000000047)	Level 0 - 5U | **AtRBCS2B**	https://www.addgene.org/50290/	5UTR	[7]
pICH41432<br>MoClo Plants Part Kit<br>Addgene (Kit #1000000047)	Level 0 - 3U + terminator | **tOCS**	https://www.addgene.org/50343/	3UTR+TERM	[7]

pICH44300<br>MoClo Plants Part Kit<br>Addgene (Kit #1000000047)	Level 0 - 3U + terminator | **tAtAct2**	https://www.addgene.org/50340/	3UTR+TE<br>RM	[7]
pICH71411<br>MoClo Plants Part Kit<br>Addgene (Kit #1000000047)	Level 0 - 3U + terminator | **tRBCS3C**	https://www.addgene.org/50345/	3UTR+TE<br>RM	[7]
pICH71431<br>MoClo Plants Part Kit<br>Addgene (Kit #1000000047)	Level 0 - 3U + terminator | **tATPase**	https://www.addgene.org/50344/	3UTR+TE<br>RM	[7]
pNK1198	Level 0 - 3U + terminator | **tHSP18.2**	https://benchling.com/s/seq-aw4qwtvqbIs9HeZ40NTA?m=slm-bYfZI7MnwrjyEz63vfML	3UTR+TE<br>RM	[18]

Table S3. Primers used in this study.

Primer name	Sequence
pr748	atgcGGTCTCaacatGGAGataggcaaccgtggacttcttcactagctcatcgagatagcaatgccactagctaatttcttacgttgtatcttatttgttttactttggggcatgacatggttaaaccccatcaaagagaaagtgtttcatccactagtcaatatacgaattcattcgaatttatccctgtaaatcctag
pr749	atgcGGTCTCaACAAtgaggtggtcgaatgattccaggtttttttttttttttttttttttgctgtttattacaccagttcatcctaagaactaggatttacagggataaattcgaatgaattcgtatattgactagtggatgaaacactttctctttgatggggtttaaccatgtcatgccccaaagtaaaacaaataa
pr750	atgcGGTCTCaacatctcaataaactaaagctaccaattaccacaatatagtcttTcatatccacttagatataaaagataaaagtaaacaaatattaaatttcatatgcacgcataggaaactcatgatcttatcttttaaatagacatctagttttcttaggttataaatagacattttgtcctagaacttcttcact
pr751	attaccacaatatagtcttTcatatccacttagatataaaagataaaagtaaacaaatattaaatttcatatgcacgcataggaaactcatgatcttatcttttaaatagacatctagttttcttaggttataaatagacattttgtcctagaacttcttcactactaaaacctagcctcaaATGTTGTtGAGACCgcat
